# Shifting the Timeline of Esophageal Adenocarcinoma: The Interplay of Lifestyle, Comedication, and Genetics

**DOI:** 10.1002/ueg2.70254

**Published:** 2026-07-01

**Authors:** Alexander Link

**Affiliations:** ^1^ Department of Gastroenterology Friedrich‐Alexander University Erlangen‐Nürnberg Medical Campus Upper Franconia Klinikum Bayreuth Bayreuth Germany; ^2^ Molecular Gastroenterology and Microbiome Research Unit Institute for University Teaching and Research Medical Campus Upper Franconia Friedrich‐Alexander University Erlangen‐Nürnberg Bayreuth Germany

Over the past several decades, the incidence of esophageal adenocarcinoma (EAC) has increased dramatically across Western populations. Although obesity, gastroesophageal reflux disease (GERD), smoking, and dietary factors are well established, important questions remain regarding the interplay between inherited susceptibility and environmental exposures. While isolated risk factors are frequently evaluated, comprehensive analyses integrating genetic, clinical, and lifestyle determinants remain scarce. The importance of genetic susceptibility was recently highlighted by a large‐scale genome‐wide association study (GWAS) that established a polygenic risk score (PRS) capable of stratifying EAC risk across populations [1].

In this context, Koch et al. [2] provide a significant contribution by systematically examining the relationship between genetic predisposition, lifestyle factors, medication use, and age at EAC onset. In a multicenter Central European cohort comprising 1742 patients with EAC, the investigators combined detailed questionnaire‐based assessments of pre‐diagnostic exposures with the validated PRS. Consistent with prior evidence, GERD and smoking were associated with an earlier age at EAC onset. Importantly, a high PRS emerged as a robust, independent predictor of earlier disease manifestation, whereas body mass index (BMI) showed no significant association with age at onset.

Previous studies have associated regular physical activity with a reduced risk of EAC [3]. The current analysis extends these observations, linking physical activity, together with diets rich in fruits, vegetables, and fish, to a later age at diagnosis. Particularly intriguing is the observation that even among individuals within the highest PRS tier, non‐smoking status and lower meat consumption were associated with a significantly delayed disease onset. While causality cannot be inferred from these cross‐sectional data, the findings strongly suggest that adverse genetic risk may be partially mitigated through behavioral factors, reinforcing lifestyle interventions as a cornerstone of primary prevention.

The analysis of medication use also provides valuable insights. Given the central role of reflux‐mediated inflammation in esophageal carcinogenesis, the association between proton pump inhibitor (PPI) use and delayed disease onset is biologically plausible and aligns with evidence supporting a protective role of acid suppression. Perhaps the most provocative finding is the apparent synergistic benefit associated with combined PPI and acetylsalicylic acid (ASA) therapy, which demonstrated the strongest association with delayed EAC onset, whereas isolated ASA use did not achieve statistical significance. However, a potential caveat in this regard is the indication for ASA treatment. In this cohort, ASA was prescribed primarily for cardiovascular conditions rather than primary chemoprevention. This introduces substantial potential for confounding by indication, meaning prospective studies designed specifically to evaluate chemoprevention will be required before these findings can alter standard clinical practice.

Furthermore, subgroup analyses revealed that PPI use showed no significant protective association in the cohort of early‐onset EAC. This suggests that early‐onset disease may be more rigidly driven by inherited genetic factors than by modifiable inflammatory pathways, highlighting the possibility that future preventive strategies may need to be tailored dynamically according to both genetic risk and age.

An additional observation of profound clinical relevance is that only approximately 25% of patients reported having a known history of Barrett's esophagus, which may develop independently of the classical Barrett's esophagus–adenocarcinoma sequence. This finding reinforces a longstanding dilemma: the vast majority of sporadic EAC patients fall entirely outside existing surveillance programs. Consequently, preventive approaches relying exclusively on Barrett's identification are insufficient to substantially reduce the overall disease burden [4, 5]. Integrating genetic profiling with lifestyle‐based risk assessments could offer a valuable complementary strategy for identifying at‐risk individuals beyond traditional surveillance cohorts.

Several limitations merit consideration. The study relies on retrospective self‐reported data susceptible to recall bias, and uses age at diagnosis as a proxy for biological disease onset. Additionally, while the marked male predominance (88.35% male vs. 11.65% female) accurately reflects real‐world epidemiology, the small number of female participants limits the statistical power to explore sex‐specific determinants—an important unmet need given emerging evidence of hormonal influences on EAC susceptibility.

In summary, Koch et al. provide compelling evidence that genetic susceptibility and environmental exposures act in concert to dictate the timing of EAC manifestation. Crucially, the finding that favorable lifestyle choices, alongside PPI and ASA medication, delay onset even among genetically high‐risk individuals provides a strong rationale for future risk‐stratified prevention strategies. As the incidence of EAC continues to rise, some of these objectives align closely with currently available international guidelines regarding lifestyle modifications [6, 7]. Conversely, other approaches, in particular, dual chemoprevention remain debatable and await prospective data to drive the field further.

## Conflicts of Interest

The author declares no conflicts of interest.

## Data Availability

Data sharing not applicable to this article as no datasets were generated or analysed during the current study.
